# Supplementary material for: Fenpicoxamid-Imprinted Surface Plasmon Resonance (SPR) Sensor Based on Sulfur-Doped Graphitic Carbon Nitride and Its Application to Rice Samples
Source: Micromachines (Basel). 2023 Dec 19;15(1):6. doi: 10.3390/mi15010006 (PMC10820838; doi:10.3390/mi15010006)
Supplement: Supplementary file 1 [file micromachines-15-00006-s001.zip › micromachines-2766742-supplementary.pdf]

Supplementary Materials

Fenpicoxamid-Imprinted Surface Plasmon Resonance (SPR) Sensor Based on Sulfur-Doped Graphitic Carbon Nitride and Its Application to Rice Samples

Şule Yıldırım Akıcı [1], Bahar Bankoğlu Yola [2], Betül Karslıoğlu [3], İlknur Polat [1], Necip Atar [4] and Mehmet Lütfi Yola [1,*]

[1] Department of Nutrition and Dietetics, Faculty of Health Sciences, Hasan Kalyoncu University,
Gaziantep 27000, Turkey; sule.yakici@std.hku.edu.tr (Ş.Y.A.); ilknur.polat@hku.edu.tr (İ.P.)
[2] Department of Engineering Basic Sciences, Faculty of Engineering and Natural Sciences, Gaziantep Islam Science and Technology University, Gaziantep 27000, Turkey; bahar.bankogluyola@gibtu.edu.tr
[3] Department of Gastronomy and Culinary Arts, Faculty of Tourism, Hasan Kalyoncu University,
Gaziantep 27000, Turkey; betul.kokay@hku.edu.tr
[4] Department of Chemical Engineering, Faculty of Engineering, Pamukkale University, Denizli 20160, Turkey; natar@pau.edu.tr
* Correspondence: mlutfi.yola@hku.edu.tr; Tel.: +90-3422118080; Fax: +90-3422118081

Sensitivity

$$LOQ = 10.0\ S / m$$

$$LOD = 3.3\ S / m$$

S: Standard deviation of the intercept and m*:* Slope of the regression line

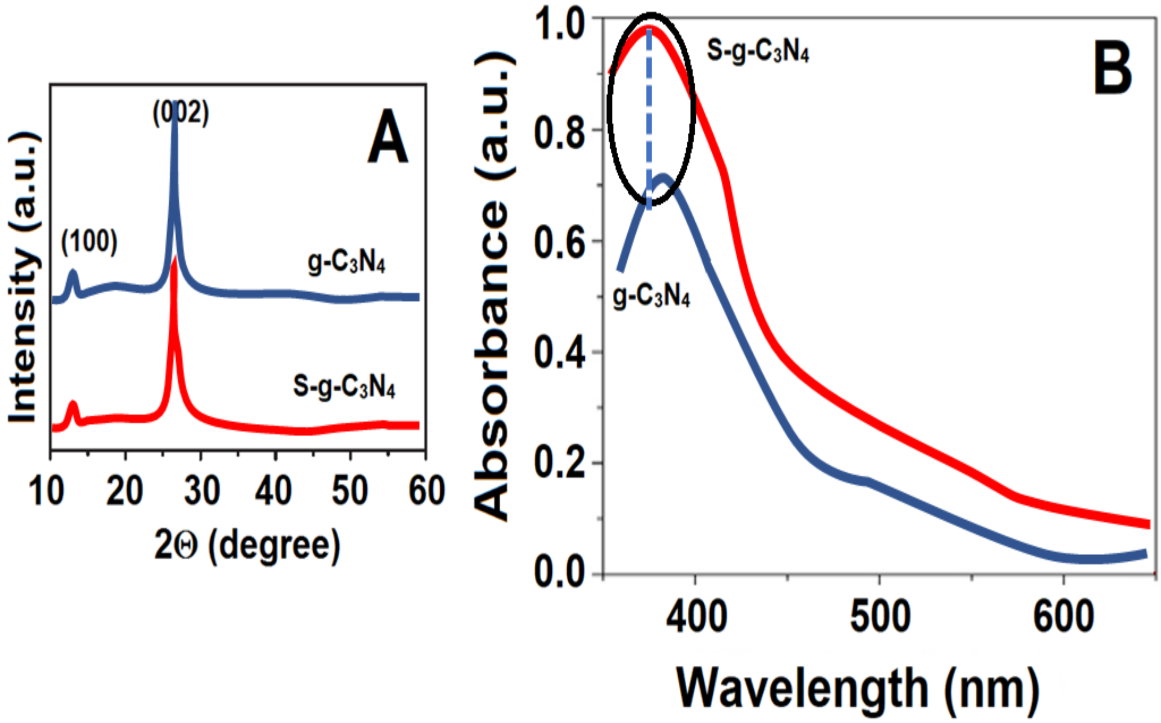

Figure S1. (A) XRD patterns and (B) UV-Vis spectra of S-g-C_3N_4 and g-C_3N_4.

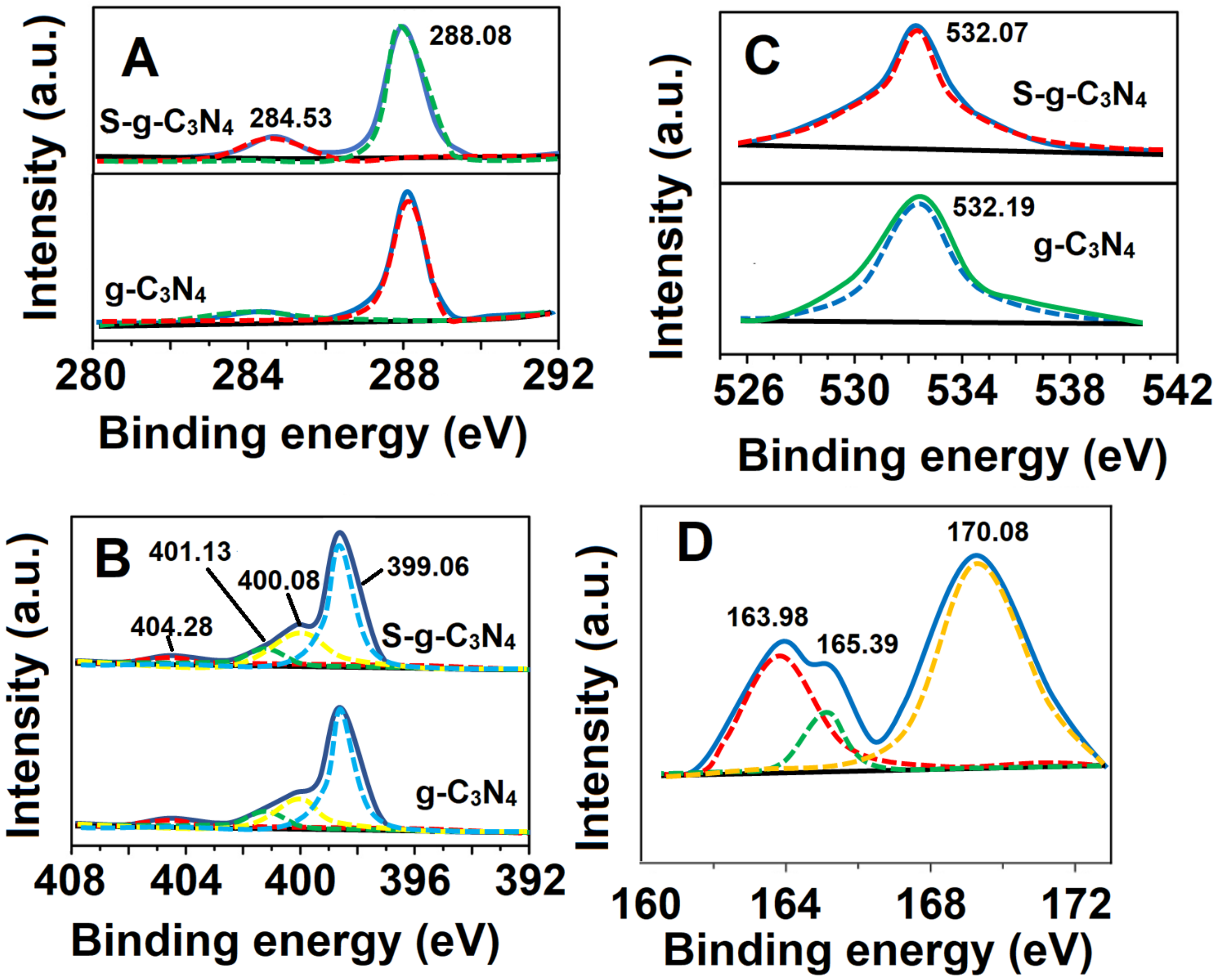

Figure S2. XPS spectra (A) C1s, (B) N1s (C) O1s of S-g-C_3N_4 and g-C_3N_4, (D) S2p spectrum of S-g-C_3N_4.

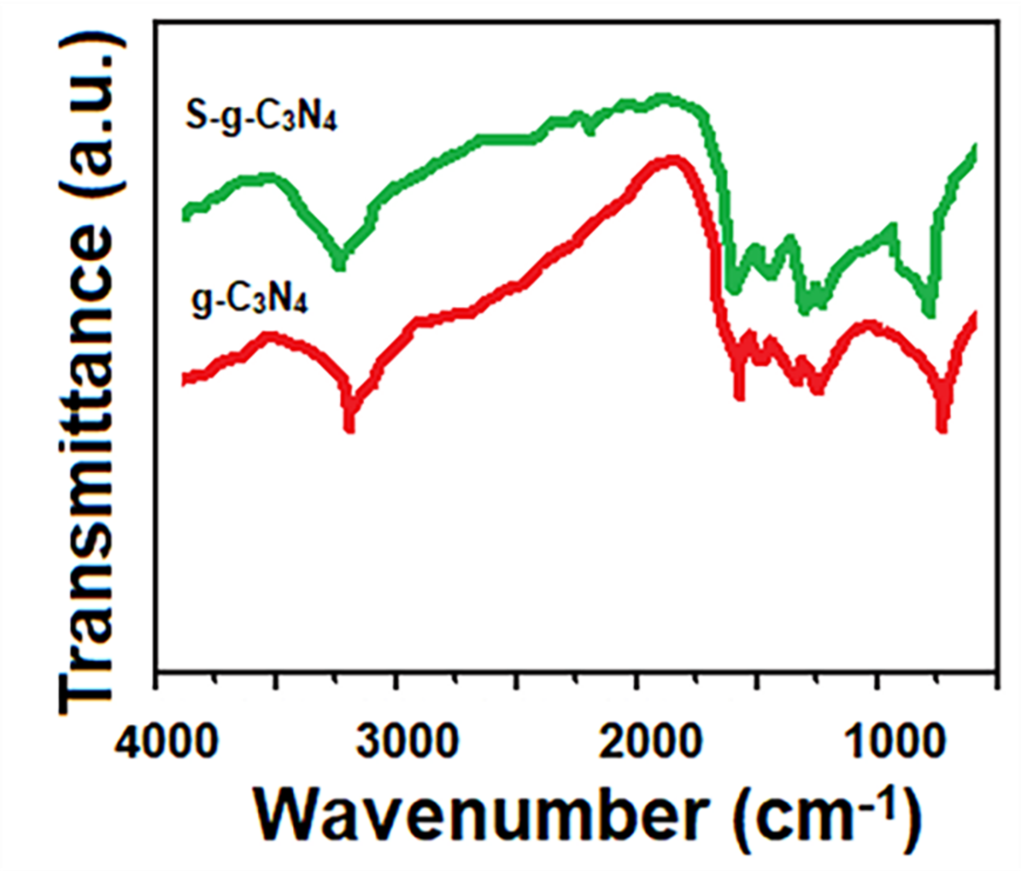

Figure S3. FTIR spectra of S-g-C_3N_4 and g-C_3N_4.

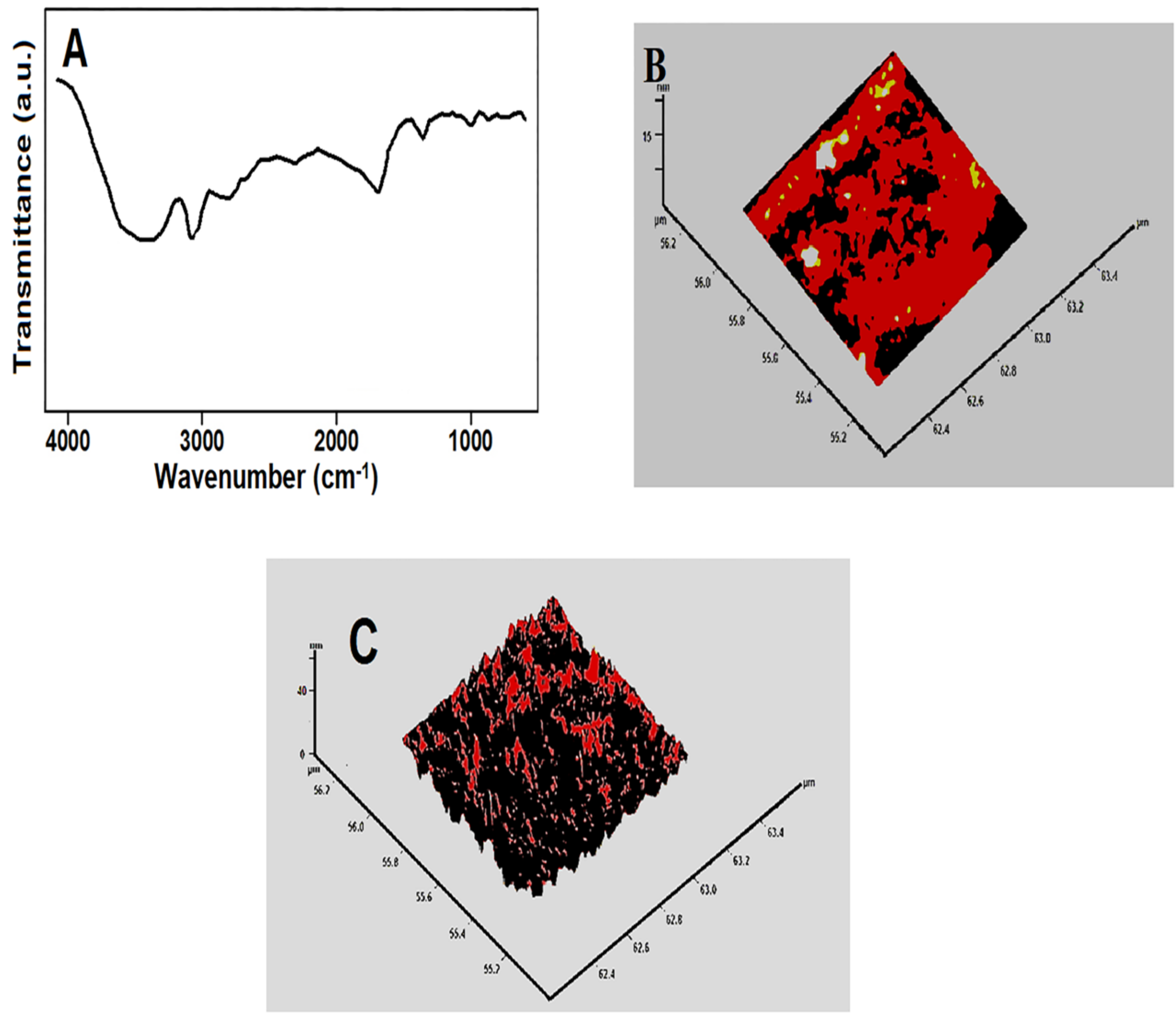

Figure S4. (A) FTIR spectra of FEN imprinted SPR chip based on S-g-C_3N_4; AFM images of (B) bare SPR chip and (C) FEN imprinted SPR chip based on S-g-C_3N_4.

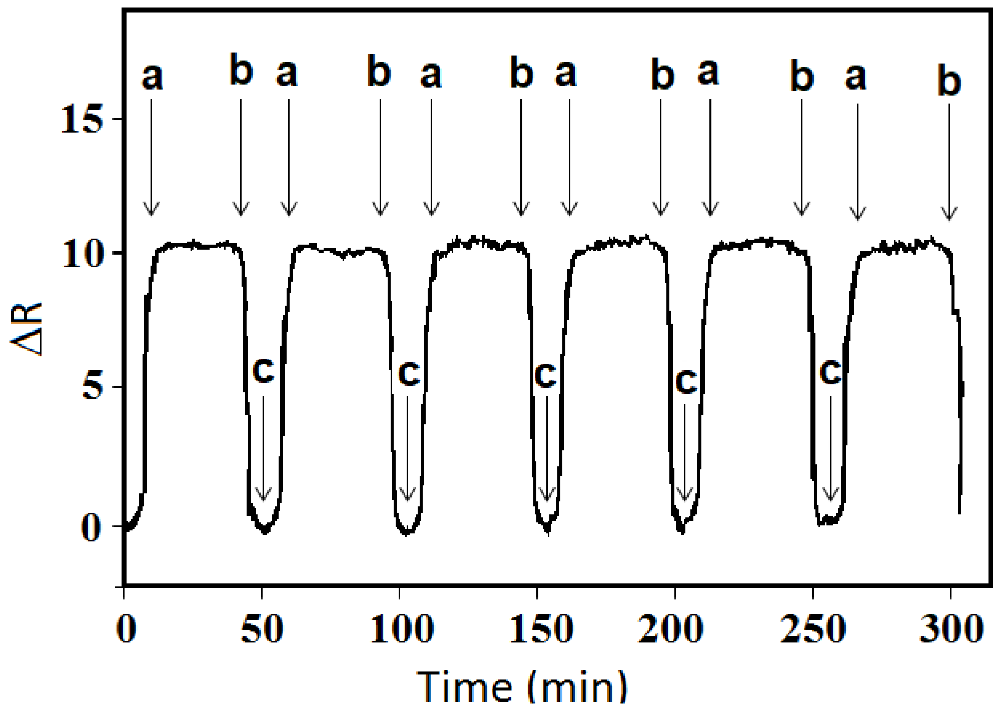

Figure S5. Repeatability of MIP/S-g-C_3N_4/SPR chip in presence of 10.0 ng L^{-1} FEN including pH 6.0 PBS at 25 °C. (a) adsorption; (b) desorption; (c) regeneration.

Table S1. Recovery results of FEN in rice grain samples (n=6)

**Added FEN ($ng\ L^{-1}$)**	**Found FEN ($ng\ L^{-1}$)**	***Recovery (%)**
-	0.17 ± 0.05	-
2.00	2.18 ± 0.04	100.46 ± 0.05
4.00	4.19 ± 0.07	100.48 ± 0.01
6.00	6.16 ± 0.02	99.84 ± 0.03

*Recovery = Found FEN, ng L^{-1} / Real FEN, ng L^{-1}

Table S2. k and k′ values of FEN imprinted SPR chips (MIP/S-g-C_3N_4/SPR and NIP/S-g-C_3N_4/SPR) (n=6)

	**MIP**		**NIP**		
	**ΔR**	**k**	**ΔR**	**k**	**k′**
FEN	10.0 ± 0.02	-	0.20 ± 0.02	-	-
UK-2A	1.00± 0.01	10.00	0.10± 0.06	2.00	5.00
AA	0.50± 0.04	20.00	0.05± 0.03	4.00	5.00

Analyte concentrations: 10.0 ng L^{-1} FEN, 1000.0 ng L^{-1} UK-2A and 1000.0 ng L^{-1} AA
$k = \Delta R_{FEN}/\Delta R_{\text{interfering chemical}}$ and $k' = k_{MIP}/k_{NIP}$.
